# Specialized Bioactive Microbial Metabolites: From Gene to Product

**DOI:** 10.1155/2015/276964

**Published:** 2015-08-05

**Authors:** Flavia Marinelli, Olga Genilloud, Victor Fedorenko, Eliora Z. Ron

**Affiliations:** ^1^Department of Biotechnology and Life Sciences, University of Insubria and The Protein Factory, Interuniversity Centre Politecnico di Milano, ICRM CNR Milano and University of Insubria, Varese, Italy; ^2^Fundación MEDINA, Health Sciences Technology Park, Granada, Spain; ^3^Department of Genetics and Biotechnology, Ivan Franko National University of Lviv, Ukraine; ^4^Department of Molecular Microbiology and Biotechnology, Tel Aviv University and MIGAL Galil Research Center, Israel

Natural products continue to play an important role in the discovery of new therapeutic candidates. Over the past 30 years, natural products or their derivatives have accounted for 60% of new anticancer agents and almost 75% of all new antibacterial molecules. One hundred natural products and natural product-derived substances were being evaluated in clinical trials or were being registered at the end of 2013. Bioactive molecules have been isolated from many terrestrial and marine organisms, including plants, marine invertebrates, and microorganisms, the latter being the source selected more often for pharmaceutical drug discovery programs. Microorganisms (traditionally filamentous actinobacteria and fungi, but more recently cyanobacteria, microalgae, and myxobacteria) are one of the most prolific sources among living organisms for the production of bioactive molecules. Exploitation of their specialized (commonly named secondary) metabolism has guaranteed for decades the discovery of novel antibiotics and other compounds with unprecedented chemical characteristics and biological properties not existing in screening libraries of synthetic compounds.

Despite the lack of big pharma interest in addressing the topic in the last decade, microbial products continue to represent today one of the most interesting sources for the discovery of novel drugs and research in the field is currently benefiting from progress that has been made in other related fields (microbial ecology, metagenomics, metabolomics, or synthetic biology), fields which have provided a deeper understanding of the microbiome and thus the development of new tools to foster the discovery of novel compounds. A wealth of microbial gene clusters that encode novel biosynthetic pathways (or interesting variants of those already described) to bioactive microbial products is being unveiled with the ever increasing number of sequenced microbial genomes. Whereas the difficulty to discover and develop in a reasonable time and acceptable cost new products from microbial sources has been widely recognized, recent advances in gene mining and heterologous expression, knowledge on regulatory networks, new analytical deconvolution, and chemical characterization tools are opening new avenues in the field of microbial product discovery.

This special issue includes five papers (three reviews and two research papers) addressing diverse aspects related to the understanding and eventually facing the current bottlenecks in the process of microbial product discovery and development.

One of the papers is a review tracing the status on antibacterial discovery and development from microbial sources. Nowadays we are all aware that bacterial resistance to all currently used antibiotics has emerged for both Gram-positive and Gram-negative bacteria. This threatening situation urgently calls for a concerted international effort among governments, the pharmaceutical industry, biotechnology companies, and the academic world to react and support the development of new antibacterial agents. The authors of this review, which include the editors of this special issue, after an introduction of the medical needs and the mechanisms of antibacterial resistance, investigate those screening ingredients (i.e., how to build microbial product libraries, methods for cultivation and extraction, the need of chemical dereplication for an early elimination of already known molecules, and tools for strain selection) that are nowadays crucial for discovering novel antibacterials. An overview of the current fermentation technology used to produce specialized metabolites and a detailed analysis of the chances for genetically improving the producing microbes in the postgenomics era are following. For the majority of antibiotics, including those recently marketed, the only feasible supply process continues to be fermentation, total synthesis being too complicated or too expensive. Thus, manipulating and improving microbial strains and their growing conditions remain the main tools to reduce production volumes and costs and guarantee quality and reproducibility of the drug bulks.

A second review by researchers from Brazil is focussing on microalgae diversity exploitation for discovering and producing interesting specialized metabolites endowed with anti-inflammatory, antimicrobial, and antioxidant activities. Microalgae are microorganisms that have different morphological, physiological, and genetic traits: they include prokaryotic (cyanobacteria) and eukaryotic organisms. Among the thousands of species of microalgae believed to exist, only a small number are stored in collections around the world, and it is estimated that only a few hundred are investigated for interesting compounds present in their biomass. After an analysis of the product potentialities of genera such as* Nostoc*,* Spirulina*,* Chlorella,* and* Dunaliella*, advantages and limits of their cultivation and extraction are investigated. Due to their photoautotrophic metabolism, microalgal cultivation processes need to be better understood: microalgae can become an environmentally friendly and economically viable source of compounds of interest, once their production is optimized in a controlled culture and properly constructed bioreactors.

The third review written by South Korean researchers discusses the recent trends in the research and production of violacein, which is a purple pigment produced by both natural and genetically modified bacterial strains. The bisindole violacein is formed by the condensation of two tryptophan molecules through the action of five proteins. The genes required for its production,* vioABCDE*, and the regulatory mechanisms employed have been studied within a small number of violacein producing strains. As a compound, violacein is known to have diverse biological activities, including as an anticancer agent and as an antibiotic against* Staphylococcus aureus* and other Gram-positive pathogens. Identifying the biological roles of this pigmented molecule is of particular interest, and understanding violacein's function and mechanism of action has relevance to those unmasking any of its commercial or therapeutic benefits. As usually happens with specialized metabolites, the production of violacein and its related derivatives is strictly regulated and its production is limited. To face this production bottleneck, various groups are seeking to improve the fermentative yields of violacein through genetic engineering and synthetic biology.

The two research papers completing the issue are brilliant examples of what was anticipated within the reviews as critical steps in the discovery and development of novel specialized metabolites. Interestingly, both of them are on lantibiotics, which represent an attractive option of a new class of molecules that might overcome arising resistance. Lantibiotics are ribosomally synthetized and posttranslationally modified peptides possessing potent antimicrobial activity against aerobic and anaerobic Gram-positive pathogens, including those increasingly resistant to *β*-lactams and glycopeptides. For some of them, a specific mode of action inhibiting cell wall biosynthesis (not antagonized by vancomycin) has been demonstrated, explaining the renewed interest for such chemical class of antibacterial peptides.

The paper published by the Italian group deals with an example of an efficient strategy for lantibiotic screening applied to 240 members of a newly described genus of filamentous actinomycetes, named* Actinoallomurus*, which is considered a yet-poorly exploited promising source for novel bioactive metabolites. By combining antimicrobial differential assay against* Staphylococcus aureus* and its L-form (also in the presence of a *β*-lactamase cocktail or Ac-Lys-D-alanyl-D-alanine tripeptide), with LC-UV-MS dereplication coupled with bioautography and database query, a novel producer of the potent microbisporicin complex was rapidly identified. Beside the interest in characterizing this novel producer of microbisporicin, this paper drives the attention to the relevance of the process termed dereplication, that is, the process of distinguishing those microbial extracts that contain known bioactive metabolites from those that contain novel compounds of interest, saving resources and speeding up the discovery process of novel drugs.

Finally, the last paper by a research group from China highlights the need to improve the fermentation conditions to sustain sublancin 168 production by a strain of* Bacillus subtilis*. Fermentation is the favourite way to produce this antimicrobial peptide, but the authors claim that the low yield of this stable lantibiotic, that has a broad spectrum of antimicrobial activity, has constrained its commercial application. In this specific case, the authors first screen carbon and nitrogen sources to identify key medium ingredients and then develop an experiment design approach to optimize chemical composition of the cultivation medium and temperature of incubation. The volumetric antimicrobial peptide productivity was double and the study envisages further increments that might be achieved following the developed model.

*Flavia Marinelli* *Olga Genilloud* *Victor Fedorenko* *Eliora Z. Ron*

